# Comparative analysis of quantitative trait loci for body weight, growth rate and growth curve parameters from 3 to 72 weeks of age in female chickens of a broiler–layer cross

**DOI:** 10.1186/1471-2156-14-22

**Published:** 2013-03-13

**Authors:** Baitsi K Podisi, Sara A Knott, David W Burt, Paul M Hocking

**Affiliations:** 1 The Roslin Institute and Royal (Dick) School of Veterinary Studies, University of Edinburgh, Easer Bush, Midlothian, Scotland, EH25 9RG, UK; 2 Institute of Evolutionary Biology, University of Edinburgh, King’s Buildings, West Mains Road, Edinburgh, Scotland, EH9 3JT, UK; 3 Current address: Department of Agricultural Research, Private Bag, Gaborone, 0033, Botswana

**Keywords:** QTL, Body weight, Growth curve, Epistasis, Broiler, Layer

## Abstract

**Background:**

Comparisons of quantitative trait loci (QTL) for growth and parameters of growth curves assist in understanding the genetics and ultimately the physiology of growth. Records of body weight at 3, 6, 12, 24, 48 and 72 weeks of age and growth rate between successive age intervals of about 500 F_2_ female chickens of the Roslin broiler-layer cross were available for analysis. These data were analysed to detect and compare QTL for body weight, growth rate and parameters of the Gompertz growth function.

**Results:**

Over 50 QTL were identified for body weight at specific ages and most were also detected in the nearest preceding and/or subsequent growth stage. The sum of the significant and suggestive additive effects for bodyweight at specific ages accounted for 23-43% of the phenotypic variation. A single QTL for body weight on chromosome 4 at 48 weeks of age had the largest additive effect (550.4 ± 68.0 g, 11.5% of the phenotypic variation) and a QTL at a similar position accounted 14.5% of the phenotypic variation at 12 weeks of age. Age specific QTL for growth rate were detected suggesting that there are specific genes that affect developmental processes during the different stages of growth. Relatively few QTL influencing Gompertz growth curve parameters were detected and overlapped with loci affecting growth rate. Dominance effects were generally not significant but from 12 weeks of age they exceeded the additive effect in a few cases. No evidence for epistatic QTL pairs was found.

**Conclusions:**

The results confirm the location for body weight and body weight gain during growth that were identified in previous studies and were consistent with QTL for the parameters of the Gompertz growth function. Chromosome 4 explained a relatively large proportion of the observed growth variation across the different ages, and also harboured most of the detected QTL for Gompertz parameters, confirming its importance in controlling growth. Very few QTL were detected for body weight or gain at 48 and 72 weeks of age, probably reflecting the effect of differences in reproduction and random environmental effects.

## Background

Combining growth models with QTL mapping facilitates the understanding of the genetics underlying physiological aspects of quantitative traits [1]. Whereas there are many reports of quantitative trait loci (QTL) for body weight and several for rate of growth [2-8] before sexual maturity, there is relatively little information on QTL for body weight at maturity or growth curve parameters. This is partly due to the fact that most meat chickens are slaughtered long before they reach maturity and growth curves cannot usefully be fitted to the data. Furthermore, several weights on each bird are required for reliable model fitting.

Detection of QTL influencing the parameters of the growth curve has been reported for mice [9], sheep [10], dairy cattle [11,12] and pigs [13]. QTL based on the analysis of growth curve models detects similar QTL as age-specific body weight provided the chosen growth functions fit the data satisfactorily [1]. Understanding the biology of the model parameters and their relationships can assist in developing a breeding strategy to modify the shape of the growth curve that would not be feasible with age specific QTL. It has been demonstrated that parameters of the growth curve are heritable and that the curve can be modified through selection on bodyweight at different ages [14-16]. This might be a useful strategy to minimise weight-related problems associated with rapid early growth, such as skeletal disease, for example.

A study was conducted to identify QTL for body weight, growth rate and parameters of the growth curve for chickens from 3 to 72 weeks of age of an F_2_ cross of a broiler male line and a White Leghorn layer. A Gompertz growth curve was fitted to the body weights for each individual and the estimated parameters of the growth curve for each bird were analysed. Age-specific body weights and body weight gains were also analysed for comparison with QTL for growth curve parameters.

## Results

### Trait means, variation and phenotypic correlations

Overall means, standard deviations and range of body weight at different ages and estimates of parameters of the Gompertz equation are presented in Additional file 1: Table S1. Mature body weight averaged 3.9 kg (range 2.0 – 5.8 kg); estimated age at the point of inflection of the growth curve was 64 d with a 95% confidence interval of 50–78 d.

Phenotypic correlations between body weights, growth rates and parameters of the Gompertz model are presented in Additional file 2: Table S2. Phenotypic correlations between weights at successive ages were relatively high, whereas correlations between growth rates at different ages were low, generally accounting for less than 10% of the variation in growth rate at later ages. There was a wide range of correlations among the parameters of the Gompertz equation. Surprisingly, correlations with mature weight (W_a_) were low; high negative correlations existed between T_i_ and K and between W_0_ and both K and L whereas there were large positive correlations between K and L.

### Analyses of epistasis

There was no evidence of epistatic QTL pairs for any of the growth-related traits except that a suggestive QTL pair involving chromosomes 2 and 3 was detected for growth rate from 24 to 48 weeks of age (results not shown).

### Body weight QTL

QTL for body weight at different ages are presented in Table 1. A total of 31 genome significant QTL, including those that affected different traits, were identified on chromosomes 1, 2, 3, 4, 6, 8, 11, 27, 28 and Z. Two significant QTL for body weight were detected on chromosomes 1 (at 12 weeks), 4 (at 6 and 12 weeks), and 3 (at 48 weeks). A further 25 suggestive QTL were identified for body weight at different ages. More body weight QTL were detected for the early growth stages (3 – 12 weeks of age) than for mature growth (after 24 weeks of age) (Table 1).

**Table 1:** Quantitative trait loci (QTL) for body weight (g) at different ages

**Chromosome**	**Position (cM)**	**F-ratio**^**1**^	**CI**^**2**^	**Flanking markers**	**Additive effect ± SE**	**Dominance effect ± SE**	**VP (%)**
*Body weight at 3 weeks of age, g*							
1	131	6.9^†^	65-604	LEI0068 - LEI0146	10.1±2.8	-2.0±4.0	2.3
1	505	4.6^†^	74-615	ROS0081 - LEI0079	13.6±4.8	10.4±12.8	1.4
2	298	16.1**	43-367	ADL0114 - MCW0056	15.9±3.2	16.4±5.5	6.0
4	148	8.8*	12-183	ADL0241 - MCW0180	27.8±6.7	-6.5±23.8	3.1
6	30	9.6*	0-42	ROS0003 - ADL0142	11.1±3.1	-11.1±4.9	2.6
8	63	7.6^†^	1-87	MCW0100- ROS0075	13.7±4.1	15.4±8.5	2.1
11	0	12.5**	0-10	LEI0110 - MCW0097	13.0±2.7	7.3±3.9	1.4
13	49	5.6^†^	9-71	LEI0083 - MCW0080	14.2±4.8	-19.7±11.8	1.8
Z	127	6.9^†^	0-127	LEI0111 - LEI0075	14.8±4.0	2.3±4.0	2.3
*Body weight at 6 weeks of age, g*							
1	130	16.0**	76-219	LEI0068 - LEI0146	42.4±7.5	-2.8±10.9	5.1
1	508	4.8^†^	0-606	ROS0081 - LEI0079	36.0±12.8	35.8±34.4	5.1
2	148	5.1^†^	34-370	ADL0176 - ADL0196	45.1±14.7	-34.9±42.4	1.4
2	286	13.3**	0-400	ROS0074 - ADL0114	39.2±8.1	23.7±12.6	4.2
3	47	10.4*	14-219	MCW0083-HUJ0006	45.9±10.2	11.8±18.4	3.2
3	235	5.5^†^	12-266	MCW0040-LEI0166	19.5±8.2	31.0±13.0	1.5
4	0	8.3*	0-69	ADL0317 - MCW0295	30.1±7.4	-1.7±10.9	2.5
4	161	21.5**	140-183	ADL0241-MCW0180	95.5±14.6	5.8±40.7	6.9
6	8	8.1*	0-43	ROS0062-ROS0003	27.0±9.0	**-37.4±15.2**	2.4
8	67	7.4^†^	0-87	MCW0100-ROS0075	41.5±11.5	25.3±23.0	2.4
11	0	11.1**	0-57	LEI0110-MCW0097	34.1±7.4	13.4±10.6	3.4
13	42	5.7^†^	12-71	MCW0340-ADL0225	47.2±14.0	-6.8±37.7	1.6
Z	119	9.8**	14-127	LEI0111-LEI0075	52.7±12.2	19.8±13.2	3.0
*Body weight at 12 weeks of age, g*							
1	137	12.2**	67-227	LEI0146-ADL0319	84.5±17.0	-5.5±26.3	3.7
1	525	10.2**	103-601	ROS0081-LEI0079	71.1±21.2	**129.9±46.2**	3.1
2	281	8.2*	49-290	ROS0074-ADL0114	87.0±17.1	23.0±27.4	4.0
3	39	10.1*	15-183	MCW0083-HUJ0006	91.1±20.2	-5.7±36.2	3.0
4	0	8.2*	0-177	ADL0317-MCW0295	62.0±15.2	-4.0±22.6	2.4
4	177	44.4**	155-183	ADL0241-MCW0180	207.7±22.0	15.6±44.8	14.5
6	30	6.2^†^	0-38	ROS0003-ADL0142	33.7±17.2	-5.5±26.3	1.7
8	61	11.2**	12-75	MCW0100-ROS0075	72.9±23.1	**155.7±46.0**	1.4
9	90	4.9^†^	0-120	MCW0135-ROS0030	26.2±22.2	**-127.2±45.1**	1.3
13	7	5.2^†^	0-71	MCW0340-ADL0225	48.8±18.4	54.0±32.5	1.4
Z	117	9.1*	8-127	LEI0111-LEI0075	110.0±25.9	32.2±28.3	2.7
*Body weight at 24 weeks of age, g*							
1	131	6.5^†^	109-543	LEI0068-LEI0146	91.6±25.9	26.2±37.1	2.3
1	560	5.0^†^	96-598	ADL0183-MCW0107	92.8±33.6	91.0±68.7	1.7
2	276	5.8^†^	0-297	ADL0236-ROS0074	94.1±27.7	-6.0±45.8	2.0
4	142	17.0**	19-169	ADL0241-MCW0180	379.6±65.6	-186.3±255.0	6.7
8	14	11.6**	0-86	MCW0305-ADL0258	107.3±26.4	107.5±38.2	4.4
8	87	6.1^†^	14-87	MCW0100-ROS0075	108.2±31.9	-72.6±49.1	2.2
13	70	7.1^†^	2-71	MCW0340-ADL0255	63.2±30.6	**-156.5±48.0**	2.6
27	0	9.5^†^	-	ROS0071	113.1±25.9	-2.5±35.7	2.6
Z	127	9.1**	0-127	LEI0111-LEI0075	137.7±37.6	**109.4±38.8**	3.4
*Body weight at 48 weeks of age, g*							
2	286	9.7*	59-297	ROS0074-ADL0114	141.7±34.0	-69.6±52.7	3.2
3	40	8.8*	1-199	MCW0083-HUJ0006	139.8±40.3	**162.0±74.1**	2.8
3	216	8.7*	2-226	ADL0306-ADL0237	106.1±33.9	**-190.9±52.5**	3.5
4	153	32.7**	137-183	ADL0241-MCW0180	550.4±68.0	-72.8±224.8	11.5
6	9	8.2^†^	0-35	ROS0062-ROS0003	151.5±37.3	15.8±62.6	2.6
8	24	7.8^†^	0-87	ADL0258-ADL0179	100.7±30.6	**116.1±47.5**	2.5
9	81	8.2^†^	23-103	MCW0135-ROS0030	135.1±44.7	**-194.7±87.1**	1.7
15	10	5.7^†^	0-49	LEI0083-MCW0080	115.8±39.5	117.1±70.2	1.7
27	0	15.2**	-	ROS0071	173.0±31.4	-20.3±43.7	5.2
*Body weight at 72 weeks of age, g*							
3	45	9.0*	0-215	MCW0083-HUJ0006	199.2±49.8	118.3±92.3	3.5
4	183	24.7**	154-183	ADL0241-MCW0180	324.7±46.7	-42.2±87.2	10.3
8	12	5.7^†^	0-87	ROS0021-ROS0026	120.9±36.9	29.6±54.2	2.0
27	0	16.5**	-	ROS0071	213.4±37.1	36.9±51.6	6.7
28	24	5.4^†^	0-42	ROS0085-ADL0299	128.6±48.9	**-194.6±93.4**	1.9

^1^Significant at 0.05 (*) and 0.01 (* *) levels experiment-wide, and (^†^) suggestive.

^2^ CI = 95% confidence interval.

The position on the chromosome for QTL at 3, 6, 12, 24, 48 and 72 weeks of age with supporting F-values, additive and dominance effects and the proportion of phenotypic variance (VP) explained by the QTL. Dominance effects in bold are >2 SE (*P* < 0.05).

The largest proportion of the phenotypic variation explained by a QTL was 14.5% for 12 week body weight at 177 cM on chromosome 4 (Table 1). The contribution of most of the QTL varied across ages e.g. the chromosome 4 QTL contribution peaked just before sexual maturity that occurred at an average of 19 weeks [17]. Several QTL affected body weight at successive ages, as expected from the phenotypic correlations (Additional file 2: Table S2). The proportion of phenotypic variation explained by significant and suggestive QTL for body weight at 3, 6, 12, 24, 48 and 72 weeks respectively was 23, 43, 39, 28, 35 and 24%.

Most of the significant QTL had positive additive effects implying that the alleles from the broiler line increased body weight. Dominance effects were generally not significant and in cases where they were significant, had negative values. A QTL for body weight at 48 weeks of age segregating on chromosome 4 at 153 cM had the largest additive effect (550.4 ± 68.0 g) and explained 11.5% of the phenotypic variation. The largest dominance effect (-194.7 ± 87.1 g) was for a QTL on chromosome 9 at 81 cM and the QTL effect accounted for 1.7% of the phenotypic variation for body weight at 48 weeks of age.

### Growth rate QTL

A total of 12 significant QTL for growth rate at different ages were identified on chromosomes 1, 2, 3, 4, 8 and 27 (Table 2). Two highly significant QTL for Gr3-6 were detected on chromosome 4 and two significant QTL on chromosome 3 for Gr24-48. More QTL were detected for the early growth rates and only suggestive QTL were identified for Gr24-48. One of the QTL for Gr3-6 on chromosome 4 explained the highest proportion of the phenotypic variation (7.2%) among growth rate QTL and jointly with the second QTL on this chromosome explained 10.9% of the phenotypic variation for this trait. Most QTL acted additively but some significant dominance effects were found for QTL on chromosomes 1, 8 and 3 respectively for Gr6-12, Gr12-24 and Gr24-48.

**Table 2:** Quantitative trait loci for growth rate at different ages

**Chromosome**	**Position (cM)**	**F ratio**^**1**^	**CI**^**2**^	**Flanking markers**	**Additive effect ± SE**	**Dominance effect ± SE**	**VP (%)**^**3**^
*Growth rate at 3–6 weeks of age, (g/d)*							
1	130	14.1**	95 – 237	LEI0068-LEI0146	1.48 ± 0.28	-0.06 ± 0.40	4.7
2	282	10.2**	15 – 382	ROS0074-ADL0114	1.34 ± 0.30	0.59 ± 0.49	3.3
3	49	17.2**	23 – 128	MCW0083-HUJ0006	2.11 ± 0.36	0.52 ± 0.66	5.9
3	237	7.1^†^	13 – 266	MCW0040-LEI0166	0.61 ± 0.30	**1.51 ± 0.46**	2.2
4	0	11.2**	0 - 37	ADL0317-MCW0295	1.29 ± 0.27	-0.27 ± 0.40	3.7
4	163	21.0**	140 - 183	ADL0241-MCW0180	3.33 ± 0.51	0.83 ± 1.38	7.2
6	11	7.5^†^	0 – 45	ROS0062-ROS0003	0.54 ± 0.33)	**-1.82 ± 0.54**	2.4
11	0	7.1^†^	0 – 67	LEI0110-MCW0097	1.02 ± 0.27	0.37 ± 0.39	2.2
13	57	4.5^†^	19 – 71	MCW0340-ADL0225	1.31 ± 0.42	-0.57 ± 0.88	1.4
Growth rate at 6–12 weeks of age, (g/d)							
1	578	7.5*	65 - 601	ADL0183-MCW0107	1.63 ± 0.51	2.96 ± 1.44	2.6
3	13	9.8*	0 – 158	MCW0169-MCW0083	1.48 ± 0.36	-1.16 ± 0.65	3.9
4	168	27.0**	146 – 183	ADL0241-MCW0180	3.49 ± 0.48	0.01 ± 1.16	2.6
9	97	5.6^†^	0 – 121	MCW0135-ROS0030	0.38 ± 0.37	**-2.24 ± 0.72**	1.9
27	0	10.7**	-	ROS0071	1.18 ± 0.28	0.62 ± 0.39	3.9
Growth rate at 12–24 weeks of age, (g/d)							
8	15	9.7**	7- 87	ROS0026-MCW0305	0.98 ± 0.26	**0.93 ± 0.38**	4.6
Growth rate at 24–48 weeks of age, (g/d)							
3	2	8.8*	1 – 266	MCW0169-MCW0083	0.70 ± 0.18	-0.45 ± 0.26	4.0
3	221	8.7*	3 – 236	ADL0237-MCW0040	0.35 ± 0.16	**-0.91 ± 0.24**	4.0
4	183	6.6^†^	29 – 183	ADL0241-MCW0180	0.63 ± 0.19	0.46 ± 0.36	2.9
28	42	6.1^†^	0 - 42	ROS0085-ADL0299	0.48 ± 0.16	-0.40 ± 0.24	2.7
Growth rate at 48–72 weeks of age, (g/d)							
2	365	7.8^†^	138 – 383	MCW0056-MCW0157	-2.74 ± 13.35	8.06 ± 4.33	3.7
4	12	6.0^†^	0 – 183	ADL0317-MCW0295	-0.04 ± 22.41	7.45 ± 11.85	2.7
7	92	7.6^†^	0 – 93	LEI0064-ROS0019	-7.75 ± 26.28	3.21 ± 18.35	3.6

^1^ Significant at 0.05 (*) and 0.01 (* *) levels experiment-wide, and (^†^) suggestive.

^2^ CI = 95% confidence interval.

The position on the chromosome with supporting F-values, additive and dominance effects and the proportion of phenotypic variance (VP) explained by QTL at successive intervals from 3 to 72 weeks of age for an F_2_ broiler-layer cross. Dominance effects in bold are >2 SE (*P* < 0.05).

The proportion of phenotypic variation explained by significant and suggestive QTL for Gr3-6, Gr6-12, Gr12-24, Gr24-48 and Gr48-72 respectively was 33, 15, 5, 14 and 10%.

### Gompertz curve parameter QTL

The QTL results for the lnGompertz curve parameter estimates are presented in Table 3. Except for mature weight, most QTL for growth curve parameter estimates were merely suggestive. Several QTL detected were for asymptotic body weight (W_A_) on chromosomes 2, 4, 8 and 27, and one QTL was identified for age at maximum growth, T_i_, on chromosome 11. Relatively few suggestive QTL were segregating for for K, L and W_0_, The proportion of phenotypic variation explained by significant and suggestive QTL for W_A_, Ti, K, L and W_0_ was 30, 8, 7, 3 and 5%.

**Table 3:** Quantitative trait loci (QTL) for Gompertz parameter estimates

**Chromosome**	**Position (cM)**	**F-ratio**^**1**^	**CI**^**2**^	**Flanking markers**	**Additive effect ± SE**	**Dominance effect ± SE**	**VP (%)**
*Asymptotic body weight (W*_*A*_*), g*							
2	283	12.0**	58 -295	ROS0074-ADL0114	150.3 ± 31.0	-18.9 ± 49.0	4.1
3	46	6.5†	1 - 205	MCW0083-HUJ0006	125.9 ± 37.0	71.5 ± 69.2	2.0
3	205	5.4†	2 - 236	ADL0306-ADL0237	106.1 ± 33.9	-67.9 ± 60.8	1.6
4	166	26.7**	143 - 183	ADL0241- MCW0180	352.6 ± 48.7	-137.4 ± 125.2	9.5
7	46	4.4†	0 - 93	LEI0064-ROS0019	65.37 ± 58.8	**486.2 ± 175.3**	1.3
8	13	8.6*	0 - 86	ROS0026-MCW0305	107.4 ± 28.1	74.3 ± 40.9	1.8
9	46	5.4†	21-115	ROS0078-MCW0135	83.3 ± 39.1	**-177.6 ± 74.0**	1.6
15	10	5.8†	0 - 45	LEI0083-MCW0080	108.7 ± 34.4	81.6 ± 62.6	1.8
27	0	18.7**	-	ROS0071	166.6 ± 28.0	46.7 ± 39.2	6.6
*Age at inflection (T*_*i,*_*), d*							
2	32	6.2†	8 - 376	ADL0343-ADL0176	-3.0 ± 0.9	0.1 ± 2.2	2.5
4	22	5.7†	0 -166	ADL0317-MCW0295	-2.1 ± 0.6	-0.6 ± 1.4	2.3
11	7	8.2*	0 - 63	MCW0097-ROS0111	-1.8 ± 0.5	-1.4 ± 0.8	3.5
*Rate of exponential decay (K), g/d*							
4	17	5.6†	0 -169	ADL0317-MCW0295	8.0 E-4 ± 2.4E-4	3.0 E-4 ± 4.8E-4	2.3
7	93	5.4†	27 - 93	ROS0019-ADL0180	6.0 E-4 ± 1.8E-4	-3.0 E-4 ± 2.7E-4	2.2
28	0	5.0†	0 - 42	ROS0095-ROS0085	-3.0 E-4 ± 1.8E-4	7.0 E-4 ± 2.6E-4	2.0
*Instantaneous growth rate (L), g/d*							
1	541	4.0†	0 - 600	ROS0081-LEI0079	0.002 ± 0.001	0.003 ± 0.002	1.5
4	144	4.9†	0 - 183	ADL0241-MCW0180	0.01 ± 0.0	0.01 ± 0.01	1.9
*Hatching weigh (W*_*0*_*)*_*,*_*g*							
8	78	6.2†	7 - 87	MCW0100-ROS0075	5.0 ± 1.5	-4.2 ± 2.7	2.6
13	62	4.9†	7 - 71	MCW0340-ADL0225	4.3 ± 1.4	2.3 ± 2.7	1.9

^1^Significant at 0.05 (*) and 0.01 (* *) levels experiment-wide, and (^†^) suggestive.

^2^ CI = 95% confidence interval.

The position on the chromosome with supporting F-values, additive and dominance effects and the proportion of phenotypic variance (VP) explained by QTL for an F_2_ broiler layer cross. Dominance effects in bold are >2 SE (*P* < 0.05).

### Comparisons of three QTL detection approaches

A comparison of the locations of QTL for body weight, rate of body weight gain and the Gompertz parameters is presented in Table 4. Growth rate QTL co-locate with most of the bodyweight QTL at the respective ages, as expected from the part-whole relationship of these traits. However, the number of detected QTL for growth rate is far fewer than those detected for body weight (Tables 1 and 2).

**Table 4:** Comparative positions of quantitative trait loci (QTL) for body weight, growth rates and growth curve parameters

**No**^**1**^	**Age specific weight and *growth rate***											**ln Gompertz parameter**				
	**3wk**		**6wk**		**12wk**		**24wk**		**48wk**		**72wk**	**W**_**A**_	**T**_**i**_	**K**	**L**	**W**_**o**_
			***Gr3-6***		***Gr6-12***		***Gr12-24***		***Gr24-48***		***Gr48-72***					
1	131	505	**130**	508	**137**	**525**	131	560							541	
			***130***			*578*					*365*					
2		**298**	148	**286**		**281**		276		**286**		**283**	32			
				*282*							*365*					
3			**47**	235	**39**				**40**	**216**	**45**	46, 205				
			***49***	*237*	***13***				***2***	***221***						
4		**148**	**0**	**161**	**0**	**177**		142		**153**	**183**	**166**	22	17	144	
			***0***	***163***		***168***				*183*	*12*					
6	**30**		**8**		30				9							
			**11**													
7												46		93		
											*92*					
8	63		67		61		**14**	87	24		12	**13**				78
							***15***									
9					90				81			46				
					*97*											
11	0			0									**7**			
				*0*												
Z	127			**119**		**117**		**127**								
M^2^	13		13		13		13	27	15	**27**	**27,** 28	15, **27**		28		13
			*13*		*27*					***28***						

^1^Macrochromosome number.

^2^Microchromosome number only is presented in the rows of the table (see Additional file 3: Table S3)

W_A_ = asymptotic (estimated mature) body weight, i.eW_o_*exp (L/K), g.

T_i_ = age at point of inflection, where Ti = (1/K)log(L/K), d.

K = rate of exponential decay, g/d.

L = growth rate per day at day zero, g/d.

W_0_ = hatching weight, (y –intercept), g.

The position (cM) of QTL for weight at a specific age (normal font), growth between successive ages (italic font) and Gompertz curve parameter estimates (normal font) are itemised. Genome significant (*P* < 0.05) QTL are in bold font; suggestive (chromosome significant) QTL are in regular font.

Different QTL on chromosome 8 at approximately 60 cM affected body weight at 3, 6 and 12 weeks, mature weight and growth rate between 12 and 24 weeks of age. A QTL at 12–15 cM affected body weight at 24 weeks, growth from 24–48 weeks and W_A_. This QTL region was also the only significant QTL for Gr12-24 which spans the age at the onset of sexual maturity. QTL were detected for growth rate across the different growth phases on chromosome 4, and to a lesser extent chromosome 3, except at 12–24 weeks of age corresponding to the age of onset of sexual maturity.

Most of the QTL for growth curve parameter estimates were for asymptotic body weight (W_A_) and were similar to QTL on chromosomes 2, 3, 4, 8, 15 and 27 for body weight at different ages. The suggestive QTL segregating on chromosome 4 at 144 cM for the rate parameter (L) is close to the location of the QTL detected for W_A_ at 166 cM and is similar to the QTL segregating across different ages at 142–183 cM on chromosome 4. The QTL for L at 541 cM on chromosome 1 is located in the same region flanked by markers ROS0081 and LEI0079 as body weight QTL at 3, 6 and 12 weeks of age (Table 1).

## Discussion

### No evidence for epistasis

Failure to detect significant epistasis for the evaluated traits was unexpected given that other studies working on similar traits detected epistatic QTL pairs for body weight at early growth stages and for Gompertz curve parameters [4,7,18,19]. Most approaches to detect epistasis are subject to a high level of false positive results [20] and the detection of only one suggestive epistatic QTL pair could be due to the stricter thresholds enforced in this analysis compared to those reported by other studies, the low density of marker coverage or the relatively small size of the population.

### Bodyweight QTL at Specific Ages

Body weight QTL at 3 weeks on chromosomes 1, 4, 13 and Z (Table 1) were similar to those detected earlier in a similar population raised as broilers for maximum growth [3]. In a previous report, chromosomes 1, 2, 3, 6 and 11 were shown to harbour QTL for body weight at 46 days [7] and also appear in the list of significant and suggestive QTL for weight at 6 weeks of age in this study (Table 1). The positions of the QTL for 6 week weight on chromosome 3 at 235 cM and 161 cM on chromosome 4 are similar to the QTL at 252 and 149 cM respectively on chromosomes 3 and 4 reported by Jacobsson et al. [21]. Most of the QTL for this trait are similar to those identified on chromosomes, 1, 2, 4, 6, 8, 13 and Z in a parallel broiler study [3].

QTL detected in the latter study for body weight at 9 weeks on chromosomes 1, 2, 4, 6, 8, and 13 are similar to those at 12 weeks of age. Positions for the QTL on chromosomes 4 and 13 respectively were at 177 and 15 cM in the earlier report and at 177 and 7 cM in this study. These are probably the same QTL because of the high correlations expected between body weight at 9 and 12 weeks of age. The significant 12 week body weight QTL on chromosomes 1, 4 and 27 are at similar locations as carcass weight QTL in a related broiler experiment [22].

QTL for body weight at 6 weeks on chromosomes 2, 3 (two locations), 4 and 6 were also identified at 48 weeks of age. Whereas body weight at two ages includes a part-whole component and the phenotypic correlation was only 0.52 (Additional file 2: Table S2), the results emphasise the importance of early growth in determining adult body weight.

### Growth rate QTL

QTL for Gr3-6 on chromosomes 1, 2, 3, 4 and 13 in this study were similar to those reported for growth from 2 to 4 weeks on chromosomes 1 and 2 [4,23]; and the QTL for Gr6-12 on chromosomes 1 and 3 are similar to those previously published for growth from 6 to 12 weeks [4].

### QTL for Gompertz parameters

The Laird form of the Gompertz equation is a function of initial growth rate, relative growth rate at the point of inflection (the rate of exponential decay of the relative growth rate) and body weight at time t_0_ compared to the original Gompertz model which is a function of mature body weight [24]. It was chosen to maximise the number of data points available for analysis (requiring the estimation of only 3 parameters) and provided an excellent fit to the data (results not shown).

The Gompertz parameter estimates (Additional file 1: Table S1) fall within the range reported in the literature: W_A_: 2483 – 5698 g, L: 0.0908 – 0.141 g/d, K: 0.0224 – 0.031, T_i_: 42.2 – 63 d, W_0_: 39.8 – 64 g [14,25-27]. The similar locations of significant and suggestive QTL for body weight and Gompertz parameters (Table 4) confirm assertions made earlier that these parameters are genetically determined and can be exploited to improve traits through selection [1,8].

Whereas sexual maturity occurs earlier in the male-line broilers than the White Leghorn layers used in this experiment [17], there was evidence of only one significant QTL for the point of inflection of the growth curve. The broiler allele of this QTL decreased the point of inflection by 1.8 d or 3.6 d in the homozygous state and if the three significant and suggestive effects from Table 3 are summed the effect is substantial (almost 7 d or 14 d in the homozygous state).

We chose to use a constant 14 h light (L): 10 h dark (D) photoschedule rather than use a typical 8 L:16D during rearing gradually increasing to, for example, 16 L:8D during lay starting when the birds are between 16 and 22 weeks of age. This was done in order to avoid compromising the growth of the birds by artificially photostimulating them at the same age when individuals would be at different physiological states caused at least in part by genetic differences. This practice therefore ensured that all the birds received the same photostimulation and that growth *per se* was not affected by differences in the reproductive responses to increasing photoperiods. It also ensured that growth was not constrained by limiting opportunity for *ad libitum* feed consumption to only 8 h per day. Significant QTL for mature body weight (W_A_) were detected on chromosomes 2, 4, 8 and 27 while suggestive QTL were detected on chromosomes 3, 7, 9 and 15. Asymptotic or mature body weight QTL have been reported by Le Rouzic et al. (2008) on chromosomes 2 and 27 and additional QTL that were not detected in our study on chromosome 1, 6 and 11 [7]. A similar QTL for age at the point of inflection was detected on chromosome 11 in both studies whereas suggestive QTL for this trait on chromosomes 2 and 4 contrast with significant QTL on chromosomes 1, 12 and 20 [7]. No genome wide significant QTL were detected for W_0_, K or L. W_0_ may be determined more by egg size and therefore maternal QTL than by QTL inherited by the chick. Genetic variation for K and L may exist but be explained more by the other parameters, particularly mature weight. Changing growth rate while maintaining acceptable hatch and mature weights may be a desirable goal in commercial meat production systems and growth curves have been modified by differential selection on early growth in chickens and quail [28,29]. Selection based on growth curve parameters is therefore likely to be effective but difficult to implement because of the length of time required to obtain body weights at older ages on which to base parameter estimates. The results suggest that relatively few areas of the genome may contain the genes largely responsible for controlling growth curves suggesting that whole genome selection based on high density SNP chips could ameliorate this problem. However, in contrast to the successful selection experiments noted above, Ibanez and Blasco [30] suggest that genomic selection to change the growth curve will be difficult and require constant re-evaluation of the associations between the SNPs and the genes determining curve parameters.

### The architecture of growth QTL

Examination of Table 4 suggests that relatively few chromosomal locations affect growth to an extent that they can be detected in this population. Taking the large confidence intervals into consideration (Tables 1, 2, 3), the results suggest that there were at least 15 such locations: 2 on each of chromosomes 1, 2, 3 and 4, one each on chromosomes 6, 8, 9, 11 and Z with an additional 2 on microchromosomes 11 and 27. These are generally consistent with other experiments and provide a smaller list of QTL in the search for causative mutations compared with the list of QTL for body weight identified at different ages such as in Table 1.

Evidence of a QTL at 166 cM on chromosome 4 for mature body weight had the largest additive effect and explained the highest proportion (9.5%) of the phenotypic variation. The results generally confirm earlier observations about the critical role of chromosome 4 in controlling growth and other traits of economic importance [3,31,32].

QTL detected for growth rate intervals (Table 2) were generally similar to those for body weight at the corresponding ages (Table 1). Both the body weight and growth rate approaches identified more significant QTL than analysis of the Gompertz curve parameters but all methods identified a significant QTL for adult body weight (W_A_) on chromosomes 2, 4, and 8.

More QTL were detected for growth before sexual maturity than for later growth and suggest that genetic variation for growth is more important during early life (Tables 1 and 2). This may in part be associated with differential development of the reproductive organs and fat deposition as the birds approach sexual maturity and by changes in reproductive status with increasing age. These effects are likely to decrease the apparent importance of growth QTL whereas those QTL associated with reproductive senescence or fatness may become more important and overshadow the growth QTL identified during the rearing of the birds. The latter are more likely to be associated with growth of the lean tissue mass (muscle, bone and nervous tissues) and after peak rates of lay by QTL for fatness. Alternatively the environmental variance may become larger with age and time in the cages. These observations are consistent with the lack of correlation between W_A_ and growth from 48–72 weeks (Additional file 2: Table S2). Further research will be necessary to determine the relative role of non-growth QTL on variation in adult weight including differences in fatness.

Age specific body weight QTL were detected for each growth stage which supports earlier observations that there are different genes and gene actions involved in growth at different developmental stages [1,4,8,9,33]. Chromosomes 3, 4 and 8 had QTL involved with body weight throughout the lifetime of the birds. Chromosome 11 harboured QTL involved mainly in very early growth and those on chromosome 1, 13 and Z from 3 to 24 weeks age. QTL on chromosomes 15, 27, and 28 mainly affected growth from 24 weeks. The few QTL that were detected at older ages (48 and 72 weeks) compared to earlier periods may reflect large environmental effects, particularly with respect to individual differences in the age that egg laying started to decline or cease altogether, and the accompanying changes in fat deposition, as discussed above.

Moderately high negative correlations between early body weight and age at inflection of the growth curve are consistent with previous research on the age at the onset of puberty which showed that large body weight QTL were associated with early onset of egg laying [17]. Thus large, faster growing birds will tend to mature earlier and therefore the age at inflection of the growth curve will also be earlier than in slower growing, smaller birds.

## Conclusions

A large number of significant QTL for body weight at specific ages was detected and most of the identified QTL affected growth rate in the preceding and/or subsequent growth stages but overall a smaller number of about 15 chromosomal locations have a substantial effect on growth in this cross. Most of the detected QTL at ages before sexual maturity have been reported in other studies and the results confirm many age specific QTL. QTL influencing Gompertz parameters were detected and these QTL also overlapped with loci affecting growth and carcass traits reported in other studies. Age specific growth QTL may be associated with specific genes and gene actions that orchestrate developmental processes during different stages of growth. Some loci featured predominantly in early growth to maturity. However, there was little or no evidence for epistasis for any growth or body weight trait. Chromosome 4 explained a relatively large proportion of the observed growth variation across the different ages, and also harboured most of the detected QTL for Gompertz parameters, confirming its importance in controlling growth. Very few QTL were detected for body weight or gain at 48 and 72 weeks of age, probably reflecting the effect of differences in reproductive decline with age and the accumulation of random environmental effects.

## Methods

### Animals and husbandry

The F_2_ population was created by crossing two males and two females from the broiler male line with two females and two males from the White Leghorn line to produce an F_1_ generation that was intercrossed as described [3]. Briefly, 8 males and 32 females of the F_1_ generation were selected and mated in a balanced mating scheme to produce over 2000 F_2_ birds. The female chicks from 9 hatches were reared in floor pens and moved to individual cages (40 cm wide × 45 cm deep × 80 cm high) at 12 weeks of age. The birds were fed *ad libitum* on a conventional poultry diet and had permanent access to water. The birds were exposed to a constant photoperiod of 14 hours per day from hatch to the end of the experiment.

The body weight of each bird was recorded at 3, 6, 12, 24, 48 and 72 weeks of age. Body weights at specific ages, growth rates between successive age intervals and growth curve parameter estimates for each individual were analysed as different traits. Growth rate at each age interval was derived by dividing the body weight gained in the interval by the difference in age (d) and were denoted as Gr3-6, Gr6-12, Gr12-24, Gr24-48 and Gr48-72 respectively for the daily weight gained from 3–6, 6–12, 12–24, 24–48 and 48–72 weeks of age.

All animal husbandry and procedures were conducted under legislation to protect the welfare of animals and were licensed after ethical review.

### Growth curve modelling

The Laird form of the Gompertz curve equation as described by Aggrey [27], was fitted to data from 453 individuals that had a minimum of 4 data points after removing extreme outliers (>3 SD from the mean). The equations for deriving the parameters of the Laird form of the Gompertz curve were defined following [34] as

$$w_{t}=w_{o}e^{\left(\frac{L}{K}\left(1-e^{-\mathrm{kt}}\right)\right)}$$

where W_t_ is the body weight of a bird at time *t*, W_0_ the estimated initial hatching weight, L the rate of growth (g/d) at time t = 0 and K the rate of exponential decay of the relative growth rate. The following derived parameters were computed: age at the point of inflection, T_*i*_ where T_i_ = (1/K)log(L/K) and asymptotic or mature body weight, W_A;_ where W_A_ = W_0_.exp(L/K). The growth curves were fitted using the Genstat program (http://www.vsni.co.uk/software/genstat) and the parameter estimates of the growth curves were extracted for each bird. The model converged for all the data points analysed.The estimated parameters were not normally distributed and to approximate normality the natural logarithm (ln) of the Laird form of the Gompertz curve was adopted.

### DNA analysis and map construction

DNA was extracted from blood samples as described previously [17]. Genotyping was performed using a total of 106 microsatellite markers covering 25 autosomal linkage groups and the Z chromosome (Additional file 3: Table S3). Genetic linkage maps were constructed using the prepare, flips and fixed options of the CRIMAP programme [35].

### QTL analysis

Mapping and significance testing were conducted by the interval mapping method for QTL analysis adapted for epistasis detection in GridQTL [20] as described earlier [17]. Significance thresholds for detection of single QTL with significant marginal effects were determined through 5000 permutations [36] and 1000 bootstraps were used to generate 95% confidence intervals for the QTL positions [37]. An F value greater than the *P* > 0.05 and *P* > 0.01 experiment-wide threshold values were used to identify a significant and highly significant QTL [38] respectively. QTL that achieved an F ratio exceeding the *P* > 0.05 chromosome-wide threshold were considered to be suggestive. Significance testing for epistatic pairs used F ratio tests for model comparisons in a nested test framework following [20].

Different models with additive, dominance and parent-of-origin genetic effects with family and pen as fixed effects (hatch was confounded with pen) were evaluated in preliminary analyses. There was no evidence for a parent-of-origin effect [38] and parent of origin effects were ignored in subsequent analyses. An additive plus dominance model was evaluated for all chromosomes simultaneously except for the Z chromosome which was analysed with an additive genetic effects model. The epistasis analysis did not include the Z chromosome.

## Competing interests

The authors declare that they have no competing interests.

## Authors’ contributions

PMH, SK and DWB contributed to the planning of the experiment. PMH contributed in the data collection. BKP and PMH performed the data analysis, discussed the results and drafted the manuscript. All authors read, provided comments and agreed on the contents of the manuscript.

## Supplementary Material

Additional file 1: Table S1 Mean, standard deviation and range of body weight at different ages and estimates of parameters of the Gompertz function fitted to the individual body weights of a population of an F_2_ broiler-layer cross. Click here for file

Additional file 2: Table S2 Phenotypic correlations between weight at successive ages, growth rates between different ages and parameters of the Gomperz growth curve for flock of female chickens from an F2 cross of a male line broiler and a White Leghorn layer. Click here for file

Additional file 3: Table S3 The number of microsatellite markers, first and last marker and map length on each linkage chromosome. Click here for file
