# Supplementary material for: Comparative analysis of quantitative trait loci for body weight, growth rate and growth curve parameters from 3 to 72 weeks of age in female chickens of a broiler–layer cross
Source: BMC Genet. 2013 Mar 13;14:22. doi: 10.1186/1471-2156-14-22 (PMC3606837; doi:10.1186/1471-2156-14-22)
Supplement: Additional file 1: Table S1 — Mean, standard deviation and range of body weight at different ages and estimates of parameters of the Gompertz function fitted to the individual body weights of a population of an F2 broiler-layer cross. [file 1471-2156-14-22-S1.doc]

**Additional Table S1.** Mean, standard deviation and range of body weight at different ages and estimates of parameters of the Gompertz function fitted to the individual body weights of a population of an F_2_ broiler-layer cross

| Trait | N | Mean ± (SD) | Range |
| --- | --- | --- | --- |
| *Body weight at different ages* |  |  |  |
| 3 week weight, g | 462 | 256±7 | 112 - 409 |
| 6 week weight, g | 459 | 781±144 | 242 - 1163 |
| 12 week weight, g | 462 | 1971±278 | 1168 - 3034 |
| 24 week weight, g | 429 | 3332±434 | 2202 - 4705 |
| 48 week weight, g | 434 | 3919±534 | 2634 - 5760 |
| 72 week weight, g | 414 | 3924±566 | 2042 - 5800 |
| *Growth curve parameter estimates* | | |  |
| Mature body weight (W_A_), g | 453 | 3807±462 | 2550 - 5276 |
| Age at inflection (T_i_), d | 453 | 64±7 | 48 - 87 |
| Rate of exponential decay (K), g/d | 453 | 0.02±0.003 | 0.02 - 0.03 |
| Instantaneous growth rate (L), g/d | 453 | 0.1±0.02 | 0.06 - 0.2 |
| Hatching weight (W_0_), g | 453 | 50.2±16.5 | 10 - 98 |
