# Supplementary material for: Comparative analysis of quantitative trait loci for body weight, growth rate and growth curve parameters from 3 to 72 weeks of age in female chickens of a broiler–layer cross
Source: BMC Genet. 2013 Mar 13;14:22. doi: 10.1186/1471-2156-14-22 (PMC3606837; doi:10.1186/1471-2156-14-22)
Supplement: Additional file 2: Table S2 — Phenotypic correlations between weight at successive ages, growth rates between different ages and parameters of the Gomperz growth curve for flock of female chickens from an F2 cross of a male line broiler and a White Leghorn layer. [file 1471-2156-14-22-S2.doc]

**Additional Table S2.**Phenotypic correlations between weight at successive ages, growth rates between different ages and parameters of the Gomperz growth curve for flock of female chickens from an F2 cross of a male line broiler and a White Leghorn layer

| *Body weight at age, weeks* | | | |  |  |  |  |  |  |  |  |  |  |  |  |  |
| --- | --- | --- | --- | --- | --- | --- | --- | --- | --- | --- | --- | --- | --- | --- | --- | --- |
| 3 | - |  |  |  |  |  |  |  |  |  |  |  |  |  |  |  |
| 6 | 0.85 | - |  |  |  |  |  |  |  |  |  |  |  |  |  |  |
| 12 | 0.59 | 0.80 | - |  |  |  |  |  |  |  |  |  |  |  |  |  |
| 24 | 0.46 | 0.60 | 0.69 | - |  |  |  |  |  |  |  |  |  |  |  |  |
| 48 | 0.37 | 0.52 | 0.65 | 0.74 | - |  |  |  |  |  |  |  |  |  |  |  |
| 72 | 0.33 | 0.42 | 0.53 | 0.58 | 0.75 | - |  |  |  |  |  |  |  |  |  |  |
| *Growth rate between ages (week), g/d* | | | | |  |  |  |  |  |  |  |  |  |  |  |  |
| 3-6 | 0.70 | 0.97 | 0.81 | 0.60 | 0.54 | 0.41 | - |  |  |  |  |  |  |  |  |  |
| 6-12 | 0.24 | 0.43 | 0.89 | 0.57 | 0.57 | 0.47 | 0.48 | - |  |  |  |  |  |  |  |  |
| 12-24 | 0.10 | 0.09 | 0.03 | 0.75 | 0.42 | 0.31 | 0.08 | -0.02 | - |  |  |  |  |  |  |  |
| 24-48 | 0.00 | 0.06 | 0.14 | -0.11 | 0.59 | 0.41 | 0.07 | 0.16 | -0.27 | - |  |  |  |  |  |  |
| 48-72 | -0.04 | -0.11 | -0.12 | -0.16 | -0.28 | 0.43 | -0.13 | -0.09 | -0.12 | -0.21 | - |  |  |  |  |  |
| *Gompertz parameters (ln)* | | | |  |  |  |  |  |  |  |  |  |  |  |  |  |
| W_A_ | 0.40 | 0.48 | 0.60 | 0.80 | 0.92 | 0.90 | 0.46 | 0.54 | 0.55 | 0.40 | 0.06 | - |  |  |  |  |
| T_i_ | -0.50 | -0.65 | -0.51 | -0.10 | 0.15 | 0.30 | -0.66 | -0.27 | 0.34 | 0.34 | 0.22 | 0.27 | - |  |  |  |
| K | 0.12 | 0.44 | 0.52 | 0.14 | -0.13 | -0.31 | 0.53 | 0.44 | -0.28 | -0.35 | -0.28 | -0.26 | -0.86 | - |  |  |
| L | -0.21 | 0.18 | 0.44 | 0.17 | -0.06 | -0.24 | 0.34 | 0.51 | -0.17 | -0.28 | -0.28 | -0.19 | -0.59 | 0.90 | - |  |
| W_0_ | 0.72 | 0.37 | 0.02 | 0.13 | 0.25 | 0.34 | 0.19 | -0.25 | 0.16 | 0.21 | 0.15 | 0.34 | 0.11 | -0.54 | -0.80 | - |
|  | 3 | 6 | 12 | 24 | 48 | 72 | 3-6 | 6-12 | 12-24 | 24-48 | 48-72 | W_A_ | T_i_ | K | L | W_0_ |
|  | Body weight at age, weeks | | | | | | *Growth rate between ages (week), g/d* | | | | | *Gompertz parameters (ln)* | | | | |
