# Supplementary material for: Comparative analysis of quantitative trait loci for body weight, growth rate and growth curve parameters from 3 to 72 weeks of age in female chickens of a broiler–layer cross
Source: BMC Genet. 2013 Mar 13;14:22. doi: 10.1186/1471-2156-14-22 (PMC3606837; doi:10.1186/1471-2156-14-22)
Supplement: Additional file 3: Table S3 — The number of microsatellite markers, first and last marker and map length on each linkage chromosome. [file 1471-2156-14-22-S3.doc]

**Additional Table S3.***The number of microsatellite markers, first and last marker and map length on each linkage chromosome*

| Chromosome | Number of markers | First marker | Last marker | Map length (cM) |
| --- | --- | --- | --- | --- |
| 1 | 17 | ROS0008 | MCW0107 | 548 |
| 2 | 13 | LEI0163 | MCW0157 | 473 |
| 3 | 15 | MCW0169 | MCW0037 | 286 |
| 4 | 4 | ADL0317 | MCW0180 | 195 |
| 5 | 5 | ROS0013 | ADL0298 | 119 |
| 6 | 4 | ADL0323 | ADL0142 | 113 |
| 7 | 3 | LEI0064 | ADL0180 | 109 |
| 8 | 9 | ROS0021 | ROS0075 | 92 |
| 9 | 4 | ROS0078 | MCW0134 | 132 |
| 10 | 1 | ADL0209 | ADL0209 | - |
| 11 | 5 | LEI0110 | ROS00112 | 71 |
| 12 | 2 | ADL0240 | ADL0044 | 34 |
| 13 | 2 | MCW0340 | ADL0225 | 68 |
| 14 | 1 | MCW0123 | MCW0123 | - |
| 15 | 2 | LEI0083 | MCW0080 | 49 |
| 16 | 1 | LEI0258 | LEI0258 | - |
| 17 | 1 | ADL0199 | ADL0199 | - |
| 18 | 2 | ROS0022 | ROS0027 | 24 |
| 19 | 1 | MCW0094 | MCW0094 | - |
| 22 | 1 | ROS0073 | ROS0073 | - |
| 23 | 1 | MCW0249 | MCW0249 | - |
| 26 | 2 | ADL0285 | LEI0074 | - |
| 27 | 1 | ROS0071 | ROS0071 | - |
| 28 | 3 | ROS0095 | ADL0299 | 39 |
| z | 6 | ROS0072 | LEI0075 | 127 |
| Total | 106 |  |  | 2479 |
